# A Qualitative Study on the Effects of the COVID-19 Pandemic on Solid Organ Transplantation

**DOI:** 10.1177/15269248231212912

**Published:** 2023-11-14

**Authors:** Angie K. Puerto Nino, Fabricio Batistella Zasso, Atina Boonchit, Sabrin Salim, Raza Mirza, Joseph Ferenbok, Istvan Mucsi, Heather Boon, Gary Levy

**Affiliations:** 1 Translational Research Program, Temerty Faculty of Medicine, 7938 University of Toronto, Toronto, Ontario, Canada; 2 Doctoral Programme in Clinical Research, Faculty of Medicine, University of Helsinki, Helsinki, Finland; 3 Department of Anaesthesiology and Pain Medicine, Mount Sinai Hospital, 7938 University of Toronto, Toronto, Ontario, Canada; 4 MD Program, Temerty Faculty of Medicine, 7938 University of Toronto, Toronto, Ontario, Canada; 5 Factor-Interwash Faculty of Social Work, 7938 University of Toronto, Toronto, Ontario, Canada; 6 Ajmera Transplant Center, University Health Network, Toronto, Ontario, Canada; 7 Leslie Dan Faculty of Pharmacy, 7938 University of Toronto, Toronto, Ontario, Canada; 8 Institute of Medical Science, 7938 University of Toronto, Thornhill, Ontario, Canada

**Keywords:** organ transplantation, COVID-19 pandemic, healthcare delivery responses, qualitative research, transplant program

## Abstract

**Introduction:** Solid organ transplantation is a lifesaving intervention requiring extensive coordination and communication for timely and safe care. The COVID-19 pandemic posed unique challenges to the safety and management of solid organ transplantation. This descriptive qualitative study aimed to understand how hospital stakeholders were affected by and responded to the COVID-19 pandemic to contribute toward improved healthcare delivery responses and strategies during times of systemic strain on the healthcare system. **Methods:** One-hour-long semistructured interviews were performed in 3 cohorts: healthcare professionals (N = 6), administrative staff (N = 6), and recipients (N = 4). Interviews were analyzed using conventional thematic content analysis. Thematic saturation was reached within each cohort. **Findings:** Twelve codes and 6 major themes were identified including the Impact on Clinical Practice, Virtual Healthcare Delivery, Communication, Research, Education and Training, Mental Health and Future Pandemic Planning. Reflecting on these codes and major themes, 4 recommendations were developed (Anticipation and Preparation, Maximizing Existing Resources and Networks, Standardization and the Virtual Environment and Caring for the Staff) to guide transplant programs to optimize healthcare pathways while enhancing the best practices during future pandemics. **Conclusion:** Transplant programs will benefit from anticipation and preparation procedures using ramping-down strategies, resource planning, and interprofessional collaboration while maximizing existing resources and networks. In parallel, transplant programs should standardize virtual practices and platforms for clinical and educational purposes while maintaining an open culture of mental health discussion and integrating strategies to support staff’s mental health.

## Introduction

Solid organ transplantation is a lifesaving intervention requiring extensive coordination and communication for timely and safe care. The rise of viral diseases, such as HIV, SARS-CoV, West Nile Virus, pandemic influenza A/H1N1, Zika, Ebola, and COVID-19, have led to a temporal reduction of transplantation activity, as resources are redirected.^1^ Multidisciplinary Transplant Program teams have been created to optimize delivery of solid organ transplantation services during such times, but the COVID-19 pandemic presented new and unprecedented challenges.^1^ The emerging COVID-19 variants have exacerbated concerns about the safety and management of solid organ transplantation given their increased transmission and severity of disease.^2^

During the COVID-19 pandemic, solid organ transplantation across the world, across institutions and within institutions, has faced drastic reconfiguration and adaptation.^3–8^ A considerable number of patients on the organ waiting list had their transplants delayed during the pandemic in Canada,^9^ resulting in a decrease in national procedural transplant volumes.^10–12^ Given that in Canada, transplant procedures were projected to increase every year by as much as 5% to 10%, the reduction observed represents a significant backlog on the solid organ transplant lists.^10–12^

The COVID-19 pandemic not only impacted the clinical activity of transplant programs but also created increased stress and challenges for patients, healthcare professionals (HCPs), and administrative staff.^1^ Therefore, it provides a rich context for understanding how contextual pressures change solid organ transplantation and what may be done to optimize future preparedness. This project aimed to understand how hospital stakeholders were affected by and responded to the COVID-19 pandemic to contribute toward improved healthcare delivery responses and strategies during times of systemic strain on the healthcare system.

## Methods

### Design

This descriptive qualitative study was conducted from January 2021 to October 2021 as approved by the Research Ethics Board (20-6129.0), and the findings were reported following the COREQ (Consolidated criteria for reporting qualitative research) guidelines.^13^ One-hour-long semistructured interviews were conducted with 3 cohorts: HCPs, administrative staff, and transplant recipients. Each cohort had a specific interview guide (available upon request). The semistructured interview guides were developed by the interdisciplinary team of authors and revised based on feedback from early interviewees.

### Setting

All interviews were carried out virtually using a HIPPA/PHIPA-compliant version of Microsoft Teams. Each interview was conducted by 2 investigators, 1 as interviewer, and the other as observer. Three researchers performed the interviews in different pair groups, which along with recordings helped enhance the reliability and credibility of the data.

### Sampling

Participants were recruited using convenience sampling from several programs within a single transplant center. The inclusion criteria consisted of being a member of one of the cohorts, 18 years old or greater, and fluent in English. Potential participants from HCPs and administrative staff cohorts were first contacted via email with a recruitment banner introducing the project. Recipients were introduced to the study by a nurse practitioner from their circle of care. Potential participants who demonstrated interest received information about the project and the consent form. Written consent was obtained prior to scheduling of the interview. To guarantee confidentiality, the researcher obtaining consent and the researchers conducting interviews were different research team members. All interested participants completed the interview, except for 1 participant who contacted the team after reaching thematic saturation.

### Information Collection

Recordings were used to collect data. Participants did not receive the interview questions prior to the encounter. To ensure confidentiality, interviewers were blinded to each participant's name and job title. At the beginning of the interview, the interviewers reviewed the study information, provided an opportunity for participants to ask questions, and reobtained (verbal) consent to record the interview.

The main topics of the semistructured interviews were the responsibilities and roles of the participants in the solid organ transplantation system, current institutional protocols, the COVID-19 pandemic influence on solid organ transplant, delivery of care, mental health, safety measures used during the pandemic, communication and education strategies during the pandemic, and suggestions for improvement.

### Data Analysis

Initial transcripts of each interview were produced by Microsoft Stream. To ensure accuracy, the Microsoft Teams’ transcripts were verified and edited prior to data analysis by listening to the recordings and correcting inconsistencies to enhance reliability and credibility of the data. Transcripts were analyzed using conventional thematic content analysis.^14^ This analysis was performed using the software Quirkos 2.3.1.^15^ Four researchers coded the first 6 interviews. In the first stage of the analysis, coders individually and independently assigned preliminary codes to the data from each interview. At this stage, the group met regularly to discuss differences and came to a consensus on the final code names and definitions to ensure rigor and credibility of the analysis. Once consensus between coders was established (consensus was reached at interview 5), the group broke off into pairs and coded the remaining transcripts. This iterative process allowed narrowing the number of themes by merging them on similar topics.

### Sample Size Determination

A sample size of 5 to 7 participants was estimated for each cohort, with the final number being determined through the assessment of thematic saturation.^16^ The first set of interviews (N = 6) was used to establish themes (ideas that consistently repeat across different interviews) by coding. Thematic saturation was achieved within each cohort. Therefore, there was a unanimous decision to stop recruitment on the 16^th^ interview. Collecting data until saturation enhanced the credibility of the data.

## Results

Sixteen participants, including 6 HCPs, 6 administrative staff, and 4 recipients, were interviewed. Participants belonged to the following programs: Kidney, Pancreas, Lung, Liver, Intestine, and Transplant Infectious Disease. Seven participants were female and 9 were male.

**Figure 1.**
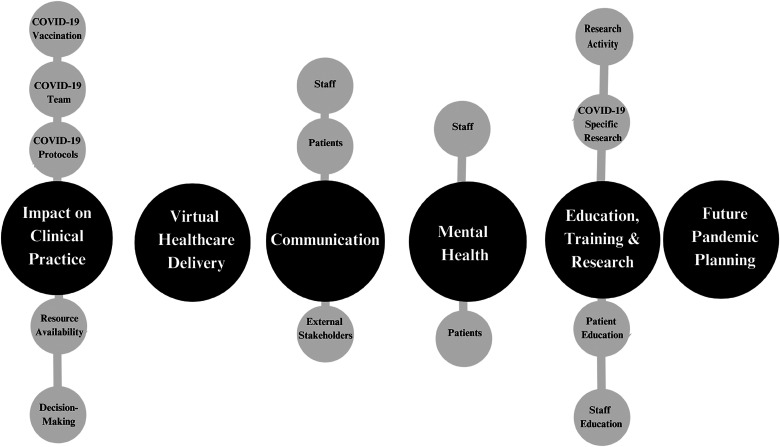
Final themes and their corresponding codes.

The initial coding process yielded 30 codes and 11 major themes. After further refinement through group discussion, there were 12 codes and 6 major themes including Impact on Clinical Practice; Virtual Healthcare Delivery; Communication; Education, Training and Research; Mental Health; and Future Pandemic Planning (**Figure 1**).

### Impact on Clinical Practice

All participants stated that the COVID-19 pandemic impacted clinical practice including care protocols and decision-making processes on the patient and administrative level. Some of the hospital staff recognized the importance of the ramping down/up approach^1^ and most participants perceived resource availability as the most pressing limiting factor of transplantation volumes: “Depending on where people were within or outside of Ontario, the availability of testing was different […] So that's been tricky, just trying to figure out how to get the data that we need on patients” (Administrative staff participant). The transplantation activity was affected by intensive care unit (ICU) and operating room availability, reduction in organ donation and decreased national and international procurement.

### Virtual Healthcare Delivery

Many participants highlighted the benefits of virtual care implementation. The most common benefits were: a significant reduction in the use of patient resources such as expenses and travel times, and a strong patients’ preference for virtual care compared to in-person appointments in favor of reducing their risk of contracting COVID-19 in the hospital. Some disadvantages were described by participants: the loss of physical examination and specific patient-cues, challenges in the integration of trainees into the virtual healthcare system, and lack of standardization in the 12 online tools used for virtual care delivery as the most frequent challenges.

Independent of the online tool used, most participants reported frustration related to technical difficulties with different tools: “We still face a lot of technical problems trying to connect with patients. They can't figure out how to use the video systems. Surprisingly, even so many months into it, that still is challenging” (Administrative staff participant). These issues are related to a lack of training on virtual healthcare delivery and coordination reported by all participants interviewed from the 3 cohorts. Some hospital staff noted virtual care delivery was more complicated by language barriers, access, and knowledge of technology for communication with racialized/marginalized/minority/immigrant groups, for example: “A significant proportion of our patients belong to racialized communities or immigrants or visible minorities where there are additional issues and don't think we're communicating to those communities efficiently” (Administrative staff participant).

### Communication With the Studied Transplant Center Staff

Various systems of communication were put in place to enable clear and concise information flow. Communication was described to be timely during the pandemic. Information regarding program status and changes was transmitted from the executive administration to the staff in the form of a quickly implemented system of mass emails, virtual meetings, and forums. The reported challenges of mass email were that messages were often fragmented, and information was not continuously streamlined, as described by 1 participant: “I think it might be nice if they opened up some more of that communication to people who were at the manager-level rather than sort of keeping everything very high [up]” (HCP participant). Standard Operating Procedures (SOPs) were also shared through these avenues but were not written as formal protocols. Participants noted that the lack of written SOPs was especially an issue at the start of the pandemic given that there were no developed pandemic plans to ensure the delivery of medical services. As new protocols were developed throughout the pandemic, fragmented communication complicated healthcare provision and administration.

### Communication With External Stakeholders and Patients

Executive staff played a crucial role in both disseminating their own findings and putting into action the information received from both national and provincial virtual calls with external organizations. These national and provincial virtual calls were the preferred method among participants of this study to learn from other programs, share experiences, and make policy decisions with an overall excellent experience: “I would participate in the meetings that were happening. At some stage it maybe even twice a week. There had to be integration throughout the province for all the organ transplant programs to sort of figure out if they were making similar policy decisions” (Administrative staff participant). All patients interviewed received information via several platforms, and in general, found these interactions helpful, intuitive, and timely. The website was found to be improved and helpful for checking updates on program status, while the phone service, Easy Call, offered by the studied transplant center, was one of the best ways to get their questions answered by their respective transplant coordinators. Conversely, HCPs found that recipients were not as well informed as they could have been.

### Education, Training, and Research

Most regular research practices, education, and training transitioned from in-person to virtual. Virtual trainee education was reportedly more accessible and wide-reaching, but less engaging and social interactions were diminished. Even though staff appreciated shifting to virtual methods for their own safety, many felt that they were inexperienced with using virtual platforms and were not given sufficient training, with one participant citing that, “One day [for] one hour would have been so much better in-person to train someone and just get this going, than sending emails, telling us which website, that was annoying. So, we did not get training” (HCP participant).

### Mental Health

The transplant staff reported increased levels of anxiety related to the uncertainty about the availability of personal protective equipment in the first wave and the possibility of transmitting COVID-19 infection to family members, stress due to concerns about the possibility of losing jobs or not being able to perform activities as expected, and fatigue and burnout were related to lack of boundaries between work and private life when working from home, and prolonged working hours. Participants that were not in supervisory positions expressed a need for more support from supervisors, citing that they did not feel supervisors sufficiently checked on staff mental health. Supervisors who were interviewed reported their efforts to address mental health issues and their belief that these efforts were successful. Several mental health resources, such as a wellness center, mental health support peer lines, a hospital intranet with stories of HCPs mental health issues, and mental health resources emails were available, but staff use was low suggesting these supports may not have been widely known or were not perceived as helpful by staff.

Two points related to culture commonly encountered in hospitals were pointed out as negative aspects of mental health. The culture of resiliency, where staff were expected to be tough, and the culture of avoiding discussion around mental health, as there was a fear that this could be interpreted “not being a hard worker” (HCP participant) by superiors and colleagues. This problem is echoed in 1 interview: “And then just feeling the pressure to ask for mental days off. I never asked for it. I’m still too afraid to even ask for anything like that unless I’m physically sick” (HCP participant).

There were opposing opinions from staff and recipients during the interviews. None of the recipients reported that their mental health was substantially affected by pandemic: “I think I've been handling it pretty well at home. Yeah, I'm lucky we have a couple of exercise machines here … I'm treasurer of a couple of organizations so I can do that online” (Patient participant). However, staff emphasized the deteriorating state of their patients’ mental health, which they attributed to patients being isolated and having increasing levels of anxiety and stress: “I think obviously patients are very stressed, especially also because surgeries are delayed. They're very worried. Many of these patients obviously are sick, and they're very worried about getting COVID-19. But I don't think we do a good job of bringing this up in appointments and talking to them, etc” (HCP participant).

### Future Pandemic Planning

The participants highlighted points they believed should be considered in preparation for future pandemics. Some of those points were the ramp-down approach in the first wave of the pandemic until the identification of the causative agent, a better understanding of the pathogenesis and its transmission were achieved, provincial and national calls as highly effective communication strategy between different transplant groups, and use virtual platforms to maximize connectivity between hospital staff, patients, and external stakeholders. The following statement underlined this idea: “…we're using the same tools to connect with patients whether they live 5 blocks away from the hospital or in Newfoundland” (Administrative cohort participant). Participants highlighted that to achieve the best virtual experience, a single standardized virtual platform should be used, better training to use these virtual platforms should be provided, and some parts of their patient's care should be handled from external facilities eliminating the need for patients to visit the hospital or clinic for all assessments and exams. Also, staff reported that the lessons garnered from previous pandemics (eg, SARS) were short term and were not carried into the COVID-19 pandemic.

## Discussion

The impact of the COVID-19 pandemic on transplantation was examined from a qualitative perspective. Transplant societies and organizations recommended an initial decrease in transplants in response to local conditions.^17^ The reduction in transplant activity witnessed globally can be attributed to limited available evidence and knowledge on the management of transplant patients; availability constraints regarding personal protective equipment and essential resources including ICU and hospital space; concerns around COVID-19 infection in transplant patients; and the impossibility of using organs for transplantation of COVID-19-positive donors.^4,5^ Transplant programs in the United States and Australia also reported implementing COVID-19 donor and recipient screening, and national and state-wide interprofessional collaboration to advise on best practices.^4,6^

The semistructured interviews revealed 6 interrelated main themes, allowing us to construct a cohesive narrative of the pandemic's impact on transplantation. The key findings were distilled into 4 recommendations for future consideration in preparation for the circumstances created by pandemics to manage and protect solid organ transplantation (**Table 1**).

**Table 1.** Recommendations for Preparation for the Circumstances Created by Pandemics to Manage and Protect Solid Organ Transplantation.

Recommendations	Considerations
Anticipation and Preparation	Ramp down during the initial phase of the pandemic
	Plan proactively the use of resources, space utilization, and patient allocation
	Promote provincial, national, and international collaboration
Maximizing Existing Resources and Networks	Use of local resources for testing
	Limit in-hospital exposure
Standardization and the Virtual Environment	Create a centralized source of written protocols
	Use a single standardized platform
	Use the virtual environment for education
Caring for staff	Promote an open culture of discussion on mental health
	Develop initiatives from leadership to check-in with staff
	Assess the effectiveness of mental health / other resources

### Recommendation 1: Anticipation and Preparation

In preparation for a pandemic's impact, solid organ transplantation should be considered an essential medical service to be maintained unless it cannot be performed safely.^7^ Ramping down of services, resource planning and interprofessional collaboration are key strategies to anticipating and preparing for a pandemic's impact on solid organ transplantation activity. First, anticipating a ramp-down of transplant activity in the initial stages of a pandemic to accommodate limited information, keep resources available for the most vulnerable transplant patients, and mitigate adverse impacts on transplant patients.^1,8^ Reports from national and international transplant programs indicate a similar response to the pandemic, as a means of mitigating potential harm and retaining vital care resources.^6,18^

Given that ICU and hospital capacity were cited as prominent factors that limited transplantation activity, proactive planning of space utilization and patient allocation may ensure transplant patients receive necessary lifesaving treatment. This strategy has been shown to be effective previously to allow no significant decline in transplant activity.^7,18^

The engagement in multidisciplinary collaboration with transplant and infectious disease specialists was an important strategy to prepare and manage pandemic-related impacts. Through quickly pooling together experts within the program, and collaborating with provincial and national groups, new knowledge, best practices, experiences, and decision-making strategies were effectively communicated. The creation of a taskforce that anticipated and advised on transplant activity aided in decision-making during the pandemic has been recommended by additional groups.^7,18^

### Recommendation 2: Maximizing Existing Resources and Networks

To limit hospital exposure, the transplant center relied on external testing resources to manage transplant patient care. Patient participants felt that using local resources for testing allowed quick transfer of health information. Given that the transplant center services a wide geographical area of transplant recipients, maximizing existing external testing resources was an effective strategy to protect and accommodate recipients who live far away. Liver transplant specialists in the United States and pediatric kidney transplant specialists in Canada supported this consideration by recommending reliable, timely, and local testing to patients as a mechanism to limit travel to transplant centers.^18,19^

### Recommendation 3: Standardization and the Virtual Environment

International transplant centers reported increased adoption of telehealth to care for transplant patients while limiting in-person visits.^20^ In Germany, the use of telehealth in live kidney donation was associated with decreased rates of acute hospitalization.^20^

Participants also reported a lack of written protocols for changes, making it difficult to keep up with dynamic responses. Changes to protocols should be written in a clear, concise, and timely fashion for staff access. Written protocols should be supplemented by a centralized source of information for staff. In clinical healthcare management, written protocols that are shared electronically promote continuity of care within multidisciplinary settings and communication within organizations.^21^

A key component of written protocols and centralized sharing of information is the standardization of virtual practices and platforms. The World Health Organization states that lack of standardization in digital health may result in fragmented communication, lost information on patient health, and subsequently poor healthcare delivery.^22^ Each transplant center may consider to use a user-friendly virtual platform best suited for their needs.^23^

In conjunction with the standardization of digital health tools, findings from this study support the importance of educating staff and patients on the platform of choice. It serves as a key strategy to mitigate and manage technological difficulties during virtual visits for HCPs and patients.^23–25^ Participants reported that spending long hours on the computer negatively impacted their mental health. While it may seem contradictory to encourage virtual platform use, staff, and patient education may optimize virtual care, thus reducing the time spent managing technological issues.

Although virtual care may have its benefits, its integration has often highlighted gross inequities and access. There was a concern that some transplant patients may have issues with accessibility to technology, internet access, and digital health literacy.^24^ Staff participants cited challenges with integrating racialized or non-English speaking communities into the virtual environment. While some of these challenges may be addressed through patient education and the integration of interpretation services into the clinical encounter, transplant centers should consider assessing the appropriateness of virtual care for each patient's needs.^26^

Another consideration to educate trainees was the quick move to the virtual environment that helped support continuity. Participants reported that the rapid switch to virtual education effectively ensured that education remained a priority during the pandemic.

### Recommendation 4: Caring for Staff

The psychological repercussions of pandemics on the mental health of frontline staff are well known, and similar effects are observed on staff working during the pandemic. As essential workers, staff were under constant pressure, working long hours, experiencing anxiety surrounding their safety, and working in environments that tended to resist discussion around mental health. Without prioritizing mental health and being provided with the appropriate support, healthcare workers will continue to experience increasingly harmful burnout levels.

To minimize mental health disturbances, it is important to promote an open culture of mental health discussion while integrating support strategies. It might be helpful if the leadership, such as hospital managers or supervisors, are the ones to establish an environment that prioritizes the protection of healthcare workers’ mental health.^27^ Furthermore, transplant programs may consider traditional and nontraditional approaches to supporting staff, including the provision of psychological services, supporting safe working environments and staff personal well-being (ie, supplying sufficient personal protective equipment, minimizing long shifts, prioritizing childcare support), and providing educational resources to promote good mental health. While different interventions may be used, it is important to monitor their impact and assess their effectiveness.

### Limitations

These findings should be evaluated with consideration of several limitations. Only a small sample of 16 participants divided into 3 cohorts from a single institution were interviewed. While this included a range of viewpoints on some transplant programs, the results may not represent the perspectives of all transplant programs within this organization. However, as thematic saturation was reached, the most important issues for transplantation service were identified. Consequently, the findings might not be directly generalizable to other transplant centers. However, the main findings can be used as a guide to discuss how they might impact on other locations and are likely transferable to settings with similar characteristics.

## Conclusion

The COVID-19 pandemic posed unique challenges to an already strained healthcare system. The experiences shared constituted a distinctive example of how healthcare delivery was affected by and responds to times of systemic stress. By collecting the perspectives of different stakeholders involved in the solid organ transplantation system, 4 recommendations were identified to serve as a guide to help optimize healthcare pathways while enhancing the best practices.
